# Novel variants of monogenic diabetes and impact of genetic diagnosis on treatment strategies

**DOI:** 10.3389/fmed.2025.1737184

**Published:** 2026-01-16

**Authors:** Ingrida Stankute, Aiste Cemerkaite, Gryte Leonaviciute, Marius Sukys, Kristina Aleknaviciene, Rasa Ugenskiene, Rasa Verkauskiene

**Affiliations:** 1 Institute of Endocrinology, Medical Academy, Lithuanian University of Health Sciences, Kaunas, Lithuania; 2 Medical Academy, Lithuanian University of Health Sciences, Kaunas, Lithuania; 3 Department of Genetics and Molecular Medicine, Medical Academy, Lithuanian University of Health Sciences, Kaunas, Lithuania

**Keywords:** MODY, monogenic diabetes, novel variants, precision therapy, WES—whole-exome sequencing

## Abstract

Monogenic diabetes (MD) is a rare form of diabetes resulting from single-gene defects. While diagnostic guidelines are well established for young patients, individuals >25 years are frequently overlooked, despite the clinical value of molecular diagnosis for personalized therapy.

**Aim:**

To evaluate genetic sequencing outcomes and their implications for treatment optimization in patients diagnosed with diabetes between 2017 and 2024 across all age groups.

**Methods:**

Among 509 individuals tested for suspected MD, 78 (60.3% female) had a confirmed molecular diagnosis. Genetic testing was performed in patients with negative pancreatic autoantibodies, a family history of diabetes, or stable hyperglycemia without insulin requirement.

**Results:**

The median age at MD diagnosis was 18.3 (4–68.1) years, with a median diabetes duration of 4.5 (0–50) years. Forty-three patients (55.1%) were diagnosed before age 25 and thirty-five (45.6%) after 25 years. GCK variants predominated in both groups (81.4% and 74.3%, respectively), followed by HNF1A, HNF4A, and HNF1B. After molecular confirmation, 75% (18/24) of eligible patients underwent actionable treatment changes according to genotype, while six did not benefit from therapy adjustment.

**Conclusion:**

These findings demonstrate a high diagnostic yield (15.3%) for MD and emphasize the need to broaden testing criteria to enable precise, gene-guided and on time treatment decisions.

## Introduction

Monogenic diabetes (MD) is a rare form of diabetes caused by one or more defects in a single gene (1, 2). Up to date, 43 genes have been linked to MD pathogenesis. Despite the fact that MD accounts for up to 5% of all diabetes cases, it is often misdiagnosed either due to phenotypical overlap with other types of diabetes or absence of clinical suspicion (1–3).

MD is a heterogeneous group of disorders, the most common being maturity-onset diabetes of the young (MODY) and neonatal diabetes. MODY has well-established diagnostic guidelines in children and young adults, however, individuals over 25 are often overlooked despite the importance of genetic diagnosis for personalized treatment and family risk assessment (4). At least 14 genes have been confirmed to cause MODY, and eight of them (*GCK, HNF1A, HNF4A, HNF1B, ABCC8, KCNJ11*, 6q24, and *INS*) have clear clinical and therapeutic implications (2, 3).

The aim of this study was to highlight the burden of undiagnosed MD and analyze genetic sequencing results and their implications for treatment optimizations during 2017–2024.

## Research design and methods

### Study participants

Overall, 509 patients were sequenced because of strong clinical suspicion of MD over a period of 8 years. Following consultation with a pediatric endocrinologist (for individuals < 18 years) or an adult endocrinologist (for individuals ≥18 years) for the diagnosis and/or management of hyperglycemia or diabetes at the diabetes reference center at the Hospital of Lithuanian University of Health Sciences (LUHS), individuals meeting at least one criterion: negative diabetes autoimmune markers, measurable C-peptide secretion with type 1 diabetes (T1D) after 5 years of diagnosis, low/no-need for insulin treatment in presumed T1D, or positive family history of diabetes in the first line relatives or multiple cases in three generations, were referred for genetic counseling. Patient's age was not considered a selection criterion. Seventy-eight confirmed MD cases were included into further statistical analysis. The main clinical data analyzed were the age at MD diagnosis, diabetes duration, family history of diabetes, treatment before and after the molecular diagnosis, fasting C-peptide concentration. Patients were included into the study after giving an informed consent. The study was carried out in accordance with the 1964 Helsinki declaration and bioethics approvals by Kaunas Regional Biomedical Research Ethics Committee (No. BE-2-5 and BE-2-51).

### Genetic analysis

#### Genetic workflow overview

**Figure 1:**
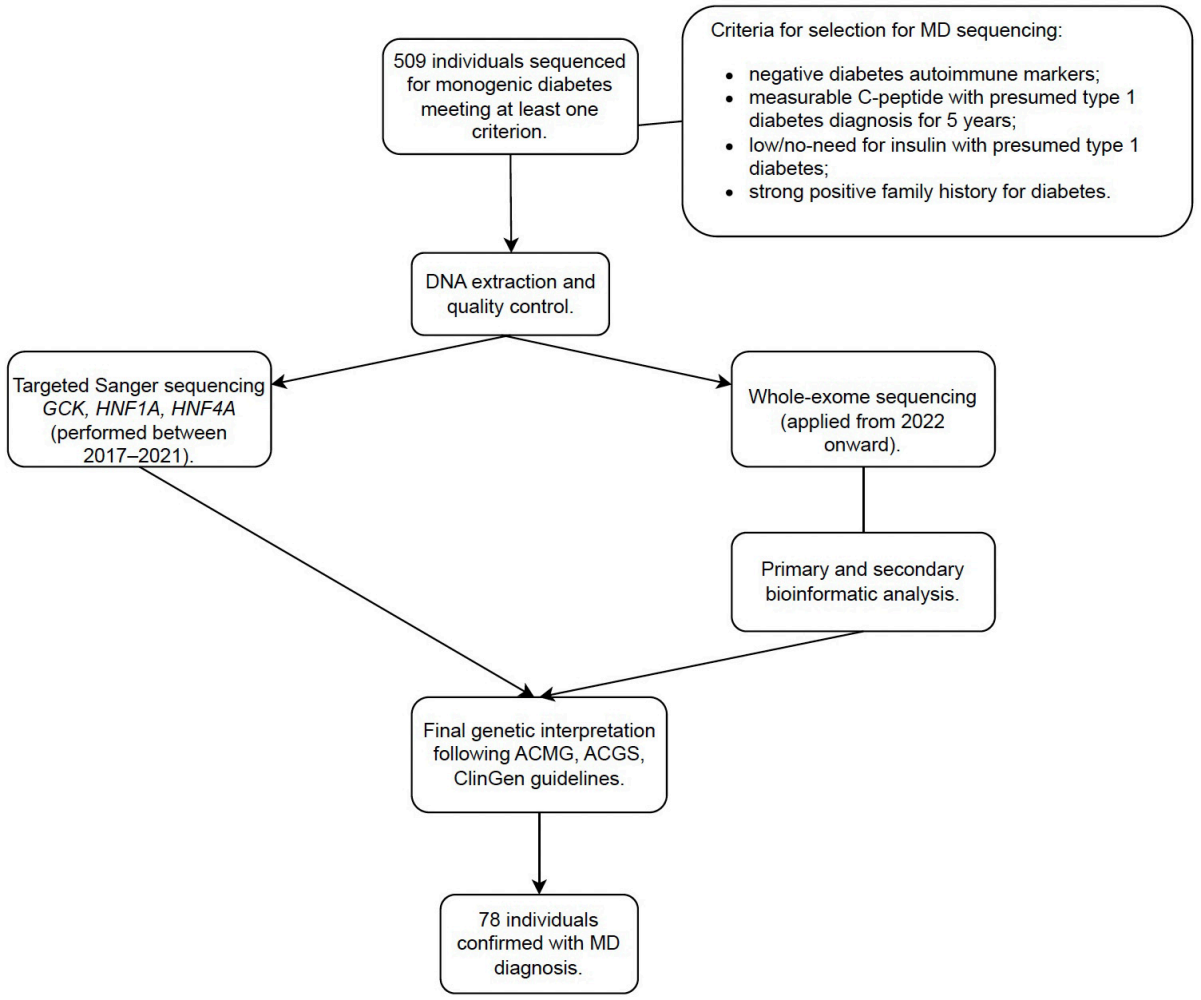
Schematic MD diagnostic workflow. MD, monogenic diabetes.

Peripheral blood samples were collected in EDTA tubes, and genomic DNA was extracted from peripheral blood leukocytes using the QIAamp DNA Mini Kit (Qiagen, Germany). DNA quantity and purity were assessed using the QIAxpert spectrophotometer (by measuring absorbance at 260 and 280 nm) (Qiagen, Germany). All samples were entered into a standardized diagnostic workflow consisting of (1) DNA extraction and quality control, (2) targeted Sanger sequencing (performed between 2017–2021) or whole-exome sequencing (WES) (applied from 2022 onward), (3) primary and secondary bioinformatic analysis, and (4) tertiary clinical interpretation following ACMG, ACGS, and ClinGen guidelines. Schematic MD diagnostic workflow is presented in Figure 1.

#### Criteria for selecting sequencing method

**From 2017 till 2021:**

Targeted Sanger sequencing was used as the first-line approach for individuals with a strong clinical suspicion of MODY:

- autosomal dominant pattern in family history,
- negative diabetes autoimmune markers,
- preserved endogenous insulin secretion (measured by C-peptide), and
- absence of features suggesting secondary or syndromic diabetes.

**From 2022 onward:**

After implementation of next-generation sequencing diagnostics, Sanger sequencing was no longer used, and WES became the sole first-line method for all newly referred patients and those who previously had inconclusive testing. WES was chosen to:

- enable broad multigene screening,
- detect rare or atypical MD forms,
- improve diagnostic yield, and
- detect SNVs, indels, and CNVs in a single assay.

#### Targeted Sanger sequencing (2017–2021)

From 2017 to 2021, targeted Sanger sequencing focused on the most common MODY-related genes: *GCK, HNF1A*, and *HNF4A*. Polymerase chain reaction (PCR) amplification of all coding exons and exon-intron boundaries was performed using gene-specific primers. PCR products were purified and sequenced bidirectionally on the Applied Biosystems 3,500 Genetic Analyzer. Chromatograms were compared with reference genomic sequences (NM_000162.5, NM_000545.8, NM_175914.4). Detected variants were interpreted using ACMG/ACGS/ClinGen guidelines. Copy number variation (CNV) was not assesed.

#### Whole-exome sequencing (2022–present)

WES was performed for all samples starting in 2022. WES was conducted by CeGaT GmbH (Tübingen, Germany), including both primary and secondary bioinformatic analysis. Protein-coding exons, adjacent intronic regions, and selected disease-relevant non-coding regions were enriched using in-solution hybridization technology. Sequencing was performed on an Illumina platform, with an average diagnostic coverage of approximately 110 × . The analysis pipeline allowed detection of SNVs, small indels, and CNVs.

Tertiary bioinformatics analysis, including annotation, classification, and interpretation of variants in the *HNF1A, PDX1, HNF4A, PDX1, HNF1B, GCK, NEUROD1, KLF11, CEL, PAX4, INS, BLK, AKT2, ABCC8, KCNJ11, APPL1, CP, CISD2, EIF2AK3, FOXP3, G6PC2, HADH, ZFP57, GLIS3, GLUD1, INSR, IER3IP1, PPARG, RFX6, SLC16A1, SLC2A2, NEUROG3, WFS1, UCP2* genes, was conducted at the Department of Genetics and Molecular Medicine, LUHS. Variants were classified based on established criteria from ACMG, ACGS, and ClinGen.

#### Variant filtering and prioritization

For WES data, initial variant filtering and prioritization were performed using the Franklin (Genoox) clinical interpretation platform. The filtering strategy included:

1. Quality-based filtering: removal of low-quality variants (coverage < 10 × , allele balance < 20%, or Phred quality < 20).
2. Frequency filtering: exclusion of variants with a minor allele frequency (MAF) >1% in gnomAD and internal population database, unless previously established as pathogenic.
3. Functional filtering: prioritization of variants predicted to have a functional impact, including missense, nonsense, canonical splice-site variants, frameshift indels, and exon-level deletions/duplications.
4. Gene-based filtering: retention of variants located within a predefined list of MODY and MD genes (full gene list provided upwards).
5. *In silico* evaluation: assessment of predicted impact using integrated tools (e.g., REVEL, SpliceAI).

Although Franklin provided automated preliminary classification, each variant was manually reviewed, and classified by medical geneticists at the Department of Genetics and Molecular Medicine, LUHS, according to ACMG, ACGS, and ClinGen guidelines. Manual curation included evaluation of segregation data (when available), reported clinical phenotypes, literature review, published functional data, and comparison with clinical databases entries.

### Statistical analysis

Data were analyzed using IBM SPSS Statistics software, version 30.0.0. Continuous variables are presented as median values with minimum and maximum in parentheses, unless otherwise specified. Categorical variables are expressed as counts and percentages. The distribution of continuous variables was assessed using the Shapiro–Wilk test. Since most variables were not normally distributed, comparisons between two independent groups were performed using the Mann–Whitney *U*-test. Categorical variables were compared using the Chi-square test or Fisher's exact test when expected cell counts were < 5. A two-tailed *p*-value < 0.05 was considered statistically significant.

## Results

### General characteristics of the cohort

Seventy-eight (15.3%) individuals out of 509 were confirmed to have MD diagnosis after genetic testing. Forty-seven (60.3%) were females. General characteristics of the cohort are presented in Table 1.

**Table 1:** General characteristics of the cohort with confirmed MD diagnosis.

**General characteristic (Median (Min;Max))**	
Age at MD diagnosis (years)	18.3 (4; 68.1)
< 25 years of age at MD diagnosis [*N* (%)]	43 (55.1%)
Hyperglycemia/diabetes duration until molecular diagnosis (years)	4.5 (0; 50)
Diabetes duration in subgroup < 25 years (years)	1 (0;14)
Diabetes duration in subgroup ≥25 years (years)	6 (0;50)
**Autoimmunity status [*****N*** **(%)]**	
Negative GAD	77 (98.7%)^*^
Negative IA2	78 (100%)
Negative IAA	75 (96.2%)^**^
Positive family history (relative with diabetes in first-line or multiple cases in three generations)	30 (38.5%)

MD, monogenic diabetes; GAD, Glutamic acid decarboxylase; IA2, tyrosine phosphatase; IAA, insulin autoantibodies. ^*^One patient with positive GAD antibodies was included in genetic testing because of strong clinical suspicion of MD (daughter was diagnosed with *HNF4A* MODY). ^**^Three patients tested positive for IAA after introduction of exogenous insulin.

### Genetic data—confirmed MD diagnosis

Overall, 78.2% (61/78) of patients were diagnosed with *GCK* mutation, causing stable hyperglycemia, 14.1% (11/78) had *HNF1A* diabetes, 3.8% (3/78)—*HNF4A*, and 3.8% (3/78)—*HNF1B*. The frequencies of affected gene by patients' age group (under 25 or ≥25 years of age) are presented in Table 2. The distribution of MD genes did not differ significantly between the two age groups (χ^2^ = 1.13, *p* = 0.77).

**Table 2:** The frequencies of affected gene by age subgroups.

**Affected gene**	**Age group**	
	<**25 years of age (*****n*** = **43)**	≥**25 years of age (*****n*** = **35)**
*GCK*	35 (81.4%)	26 (74.3%)
*HNF1A*	5 (11.6%)	6 (17.1%)
*HNF4A*	1 (2.3%)	2 (5.7%)
*HNF1B*	2 (4.7%)	1 (2.9%)

### Novel variants

Nine patient out of 78 (11.5%) were identified with novel variants in MODY genes. Three novel variants were detected in the *GCK* gene, of which 2 were familial diabetes. One novel variant was identified in *HNF4A* gene, referred as familial case. A single novel variant was found in two siblings in the *HNF1B* gene, referred due to a family history of renal anomalies. Additionally, two novel variants were detected in *HNF1A* gene. A more detailed overview of all novel variants is provided in Table 3.

**Table 3:** Description of novel variants.

**Gender**	**Age at MD diagnosis (years)**	**Affected gene**	**Variant**	**Protein change**	**Zygosity**	**Testing technique**	**ACMG variant classification criteria**	**Classification**
f	57,6	*HNF4A*	c.704G>T	p.Arg235Ile	Het	Sanger seq	PM2_supporting, PP3, PM1_supporting, PP1_strong, PP4_moderate	Likely pathogenic
m^*^	9,1	*GCK*	c.1266del	p.Phe423SerfsTer8	Het	NGS	PVS1, PM2_supporting, PP4_moderate, PS2_moderate	Pathogenic
f^**^	19,5	*GCK*	c.471_473delAGA	p.Glu157del	Het	Sanger seq	PM2_supporting, PM4_supporting, PP3, PS1_supporting, PP4_moderate	Likely pathogenic
m^**^	52	*GCK*	c.471_473delAGA	p.Glu157del	Het	Sanger seq		
m	48,7	*GCK*	c.513C>A	p.Phe171Leu	Het	Sanger seq	PM2_supporting, PM5_supporting, PP2, PP3, PP4_moderate	Likely pathogenic
f^***^	12,8	*HNF1B*	c.701dupA	p.Asn234LysfsTer60	Het	Sanger seq	PVS1, PM2_supporting, PP1, PP4	Likely pathogenic
m^***^	6,11	*HNF1B*	c.701dupA	p.Asn234LysfsTer60	Het	NGS		
f	16	*HNF1A*	c.809A>C	p.Asn270Thr	Het	Sanger seq	PM2_supporting, PM1, PP3, PP1, PP4_moderate	Likely pathogenic
f	47,9	*HNF1A*	c.1763_1781insACCGGCTCAGCCCCAGCCC	p.Thr589Profs^*^66	Het	Sanger seq	PVS1, PM2_supporting, PP4	Likely pathogenic

f, female; m, male; Het, heterozygous, seq, sequencing; NGS, next-generation sequencing.

^*^Male patient with *GCK* variant c.1266del, negative autoimmune markers, though requiring insulin injections for the treatment to reach stable glycemias, described below.

^**^Daughter and father.

^***^Siblings.

### The molecular diagnosis and treatment adjustment

Seventeen (22.1%) patients had successful treatment optimization to therapy aligned with the genetic etiology. Ten of them were patients with *GCK* diabetes: 4 discontinued insulin injections after the diagnosis, 5—discontinued Metformin, 1—discontinued Gliclazide. One patient (9 years old boy) with novel *GCK* variant continued with insulin treatment, after several unsuccessful attempts, though all pancreatic antibodies were negative, and C-peptide was normal. Seven patient with *HNF1A* diabetes were successfully switched to sulfonylurea agents: 5 from insulin, 2—from Metformin. Four patients with *HNF1A* diabetes stayed with insulin treatment, because unresponsive to sulfonylurea treatment. *HNF1B* patients did not require any treatment before or after molecular diagnosis. Two patients with *HNF4A* continued insulin therapy, and a one patient did not require any treatment. Treatment optimization according to affected gene is presented in Table 4.

**Table 4:** Treatment adjustment according to affected gene in monogenic diabetes patients.

**Affected gene**	**Treatment was appropriate (*N*)**	**Changed to first-line therapy (*N*)**	**Unsuccessful treatment optimization (*N*)**	**Total patients (*N*)**
*GCK*	50	10	1	61
*HNF1A*	0	7	4	11
*HNF4A*	1	0	2	3
*HNF1B*	3	0	0	3
Total	54	18	6	78

### Clinical factors associated with the persistence of non-first-line therapy

Main clinical characteristics of seven patients with *HNF1A* diabetes and successful treatment optimization were compared to 6 patients (4 *HNF1A* and 2 *HNF4A*) with sustained non-first-line treatment (all with insulin injections), comparison is presented in Table 5.

**Table 5:** Comparison between optimized and sustained treatment group in *HNF1A* and *HNF4A* patients.

**Variable**	**Optimized treatment (*n* = 7)**	**Sustained non-first-line treatment (*n* = 6)**	***p*-value**
Age at MD diagnosis (years)	16 (12.5; 56.3)	46 (23.8; 65.3)	0.073
Diabetes duration until MD diagnosis (years)	4 (0.1; 25)	21.5 (0.1; 50)	0.197
C-peptide at MD diagnosis^*^ (nmol/L)	0.48 (0.21; 1.04)	0.35 (0.23; 1)	0.537

MD, monogenic diabetes.

^*^Fasting C-peptide normal range 0.23-0.81 nmol/L.

## Discussion

A key strength of our study is real data from a national diabetes reference center and the inclusion of MD cases across all age groups, extending beyond the younger cohorts. We believe these findings may encourage greater attention to the genetic investigation of patients well into their 30s, 40s, and even 50s.

The frequency of *GCK, HNF1A, HNF1B*, and *HNF4A* variants were comparable in patients < 25 years and ≥25 years in our study, underscoring the importance of considering MD screening across all age groups. In pediatric cohorts, GCK-MODY typically predominates. For example, an Italian multicenter study of 172 families reported that ~80% of cases were due to *GCK* mutations, with relatively few *HNF1A* diabetes (5). Similarly, targeted sequencing in UK pediatric and young adult populations has confirmed that *GCK* and *HNF1A* together account for most MD cases (6). Studies of broader age spectrum cohorts also indicate that *GCK, HNF1A*, and *HNF4A* together account for at least 85% of MODY cases (7). Importantly, the penetrance of *HNF1A* is strongly age-dependent, with diabetes present in ~63% of carriers by age 25, ~94% by age 50, and ~99% by age 75 (8). Furthermore, population-based analyses suggest that HNF1A-MODY represents up to 70% of European cases, with the majority of affected individuals manifesting before the age of 25 (9). Taken together, these findings are consistent with our observation reinforcing the importance of offering MD genetic testing beyond pediatric and young adults' settings.

MD is frequently misdiagnosed, with many patients initially classified as having type 1 or type 2 diabetes due to overlapping clinical features and limitations of standard diagnostic algorithms, especially in patients over 25 years of age (1, 2). It is estimated that up to 80% of MODY cases are not correctly identified, resulting in significant diagnostic delays that often exceed a decade from the onset of hyperglycemia to the confirmation of a molecular diagnosis (10). Studies demonstrate that the time between the initial manifestation of hyperglycemia and genetic confirmation is prolonged, leading to extended periods of inappropriate management and missed opportunities for pathogenetically targeted therapy (1, 11–13). In our cohort, although the diagnostic interval was shorter than previously reported, with a mean duration of approximately up to 5 years from the initial diabetes diagnosis to genetic confirmation, substantial delays were still observed in some cases.

Delayed diagnosis in MD, particularly in *HNF1A* and *HNF4A*, compromises the efficacy of targeted treatments. Prolonged exposure to hyperglycemia accelerates β-cell failure through glucotoxicity, resulting in reduced endogenous insulin secretion (14, 15). Patients with *HNF1A* diabetes and longstanding diabetes exceeding a decade rarely achieve optimal glycemic targets [HbA1c < 7.5% (58 mmol/mol)] after initiating sulfonylurea monotherapy, in contrast to those diagnosed earlier who demonstrate success rates above 80% (12, 14). Diminishing β-cell function over time also necessitates adjunctive therapies despite genetically appropriate treatment. Importantly, early commencement of sulfonylureas has been associated with improved metabolic outcomes, greater preservation of insulin secretory capacity, and delayed onset of diabetes-related complications (2, 15). These findings emphasize that the therapeutic window for effective sulfonylurea monotherapy narrows considerably as diagnostic delays increase, highlighting the critical importance of early genetic identification in optimizing long-term management. A similar pattern was identified in our cohort, where individuals with a longer duration of diabetes demonstrated a reduced likelihood of achieving successful transition to sulfonylurea monotherapy, though we did not find statistical significance, likely influenced by the small sample sizes, limiting the study's statistical power.

The main limitation of our study is that comprehensive genetic testing, such as WES was not systematically performed in all patients. Instead, molecular diagnosis was largely based on targeted gene analysis, which may have led to an underestimation of the prevalence of rarer or atypical forms of MD. This approach also carries the risk of missing pathogenic variants in genes not included in the targeted panel, as well as non-coding or structural variants detectable only by broader sequencing methods. Consequently, the genetic spectrum described in our cohort may not fully capture the heterogeneity of MD. Future studies employing exome or genome sequencing across entire cohorts are warranted to provide a more complete picture of the underlying genetic architecture and to refine genotype–phenotype correlations.

The other limitaion is that this study identified several novel variants, however, no experimental functional studies (e.g., *in vitro* assays, splicing analysis, or protein function experiments) were performed to confirm their pathogenicity. This represents a limitation, as *in silico* prediction tools alone have restricted accuracy. However, segregation analysis was performed in families where additional samples were available, and segregation results contributed supporting evidence for variant interpretation. Final classification was based on a combination of ACMG/ACGS/ClinGen criteria, variant type, population frequency, predicted functional effect, and the consistency between the genotype and the patient's clinical phenotype. Nevertheless, we acknowledge that future studies including functional assays will be essential to strengthen the evidence for pathogenicity, particularly for the novel variants reported here.

Traditional screening criteria for MD—based on diagnosis before the age of 25 years, a non-obese phenotype, and a strong family history—have been widely applied increasingly recognized as overly restrictive (10, 16). Recent studies show that a substantial proportion of MD cases are diagnosed beyond the classical age threshold. In a Chinese cohort, 72.2% of patients with genetically confirmed MD were diagnosed after the age of 35, and only 38.9% had a family history of diabetes, highlighting the limitations of traditional screening criteria (17). Furthermore, a 2023 systematic review by Murphy et al. found that relying on the conventional age cutoff (< 25 years with family history) would miss about 63% of actual MD cases (16). These findings underscore the necessity to broaden current diagnostic criteria, extending genetic testing to adults diagnosed after the age of 25 who present with atypical diabetes features. Such an approach would facilitate earlier detection of MD and the timely implementation of pathogenetically appropriate therapies, thereby optimizing long-term clinical outcomes.

Finally, our data emphasize that accurate differentiation of MD from other more common types of diabetes is clinically essential, as it directly influences treatment decisions, long-term outcomes, and healthcare costs. In contrast to polygenic forms of diabetes, specific MD types have well established, gene-specific strategies. Misclassification of diabetes may lead to unnecessary or inappropriate treatment, including lifelong insulin therapy, increased risk of hypoglycemia, psychological burden, and avoidable healthcare costs. In this context, genetic testing—when applied to carefully selected patients—is cost-effective, as it enables precise diagnosis, optimization of therapy, and prevention of long-term complications related to overtreatment (2, 18, 19). Furthermore, it facilitates cascade screening of family members, allows early identification of affected relatives. Therefore, timely and accurate identification of MD is a key component of precision medicine in diabetes care, improving both individual patient management and broader family-based risk assessment.

## Conclusion

Diagnostic delays in MD remain common, contributing to extended periods of non-optimized management. Our findings underscore the clinical value of genetic testing for MD across all age groups and support its role in guiding individualized, evidence-based treatment strategies.

## Data Availability

The original contributions presented in the study are included in the article/supplementary material, further inquiries can be directed to the corresponding author.
